# The iron-sulfur cluster assembly machineries in plants: current knowledge and open questions

**DOI:** 10.3389/fpls.2013.00259

**Published:** 2013-07-24

**Authors:** Jérémy Couturier, Brigitte Touraine, Jean-François Briat, Frédéric Gaymard, Nicolas Rouhier

**Affiliations:** ^1^Interactions Arbres/Micro-organismes, Faculté des Sciences, UMR1136 Université de Lorraine-INRAVandoeuvre, France; ^2^Biochimie et Physiologie Moléculaire des Plantes, Centre National de la Recherche Scientifique-INRA-Université Montpellier 2Montpellier, France

**Keywords:** iron-sulfur, assembly machineries, iron donor, repair, scaffold proteins, carrier Proteins

## Abstract

Many metabolic pathways and cellular processes occurring in most sub-cellular compartments depend on the functioning of iron-sulfur (Fe-S) proteins, whose cofactors are assembled through dedicated protein machineries. Recent advances have been made in the knowledge of the functions of individual components through a combination of genetic, biochemical and structural approaches, primarily in prokaryotes and non-plant eukaryotes. Whereas most of the components of these machineries are conserved between kingdoms, their complexity is likely increased in plants owing to the presence of additional assembly proteins and to the existence of expanded families for several assembly proteins. This review focuses on the new actors discovered in the past few years, such as glutaredoxin, BOLA and NEET proteins as well as MIP18, MMS19, TAH18, DRE2 for the cytosolic machinery, which are integrated into a model for the plant Fe-S cluster biogenesis systems. It also discusses a few issues currently subjected to an intense debate such as the role of the mitochondrial frataxin and of glutaredoxins, the functional separation between scaffold, carrier and iron-delivery proteins and the crosstalk existing between different organelles.

## General introduction

Plants have a high iron demand, in particular in chloroplasts and mitochondria, to ensure the functionality of many vital processes such as photosynthesis and respiration. Besides, many metabolic pathways require the presence of metalloproteins including those containing iron in the form of Fe-S clusters, heme, siroheme and mono- or bi-nuclear non-heme iron centers (Johnson et al., 2005; Ye et al., 2006b). For instance, sulfur and nitrogen assimilation, chlorophyll catabolism, DNA replication and repair, ribosome biogenesis, tRNA thio-modification or co-enzyme (biotin, lipoic acid, thiamine) synthesis also rely on the functionality of Fe-S proteins (Lill, 2009; Balk and Pilon, 2011). In general, Fe-S proteins perform a wide diversity of functions ranging from electron transfer, (de)hydration reactions, radical-generation or disulfide cleavage (Johnson et al., 2005). In both eukaryotes and prokaryotes, Fe-S proteins are first synthesized in an apoform *via* the cellular translational machinery. The prosthetic group is often required for correct folding or stability of the protein. It should therefore be inserted co-translationally or immediately upon translation of the apo-polypeptides through specific assembly machineries. The precise functioning of these machineries and their regulation by environmental constraints are only poorly understood.

### Types of Fe-S clusters and interconversion

**Figure 1:**
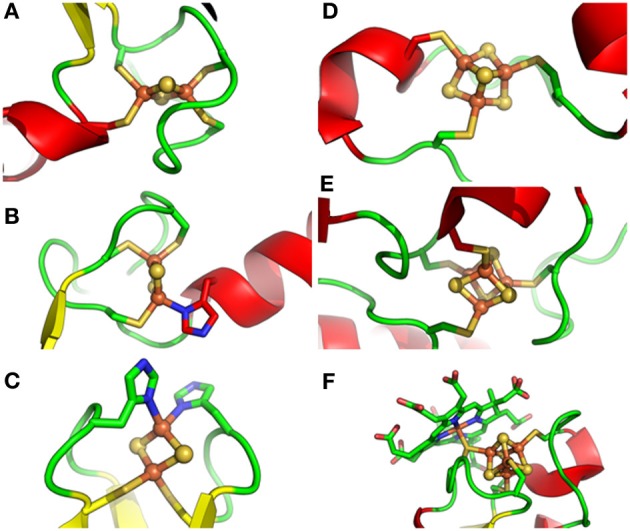
**Different types of Fe-S clusters found in proteins from photosynthetic organisms**. Schematic representation of Fe-S clusters and their ligands with sulfur, iron and nitrogen atoms colored in yellow, green and blue, respectively. **(A)**. classical [Fe_2_S_2_] ligated by four cysteines as in ferredoxin from *Cyanidioschyzon merolae*, **(B)**. NEET-type [Fe_2_S_2_] ligated by three cysteines and one histidine as in *Arabidopsis thaliana* NEET, **(C)**. Rieske-type [Fe_2_S_2_] ligated by two cysteines and two histidines as in the Rieske protein subunit of the *b*_6_/*f* complex from *Spinacia oleracea*, **(D)**. [Fe_3_S_4_] ligated by three cysteines as in *Synechocystis sp. PCC 6803* glutamate synthase, **(E)**. [Fe_4_S_4_] ligated by four cysteines as in the ferredoxin-thioredoxin reductase from *Synechocystis sp. PCC 6803*, and **(F)**. [Fe_4_S_4_] ligated by four cysteines but with one thiolate ligand serving also for siroheme as in *Nicotiana tabacum* nitrite reductase. PDB codes used for drawing these clusters using Pymol are 3AB5, 3S2Q, 1RFS, 1OFD, 2PU9, and 3B0G, respectively.

Fe-S clusters are prosthetic groups formed by iron atoms and acid-labile inorganic sulfide. In general, iron atoms are coordinated with the protein backbone *via* thiol groups of cysteinyl residues, but other more rarely encountered ligands are His, Arg, Ser or Glu residues. The most common clusters found in plant proteins are the [Fe_2_S_2_] and [Fe_4_S_4_] clusters liganded by four Cys residues, but other examples are found, as the [Fe_2_S_2_] Rieske-type clusters coordinated by 2 Cys and 2 His residues, the [Fe_2_S_2_] NEET-type clusters coordinated by 3 Cys and 1 His residues, the [Fe_3_S_4_] clusters coordinated by 3 Cys and the [Fe_4_S_4_] clusters with one Cys ligand also serving for ligating siroheme (Figure 1) (Johnson et al., 2005; Nechushtai et al., 2012). Some proteins, especially those involved in the Fe-S cluster biogenesis machineries, can assemble different types of cluster *in vitro* and the interconversion observed between these clusters might have a physiological relevance. For example, the *Azotobacter vinelandii* assembly proteins, IscU and ^(Nif)^IscA, can accommodate either a [Fe_2_S_2_] or a [Fe_4_S_4_] cluster and reversible cycling between these forms is effective *via* the reductive coupling of two [Fe_2_S_2_] clusters to form a [Fe_4_S_4_] cluster or, in the case of ^(Nif)^IscA, *via* the O_2_-induced oxidative cleavage of the [Fe_4_S_4_] (Chandramouli et al., 2007; Mapolelo et al., 2012b). Another example of interconversion has been described for the bacterial fumarate and nitrate reduction (FNR) regulatory protein. In response to elevated oxygen levels, the dimeric DNA binding form of FNR which binds a [Fe_4_S_4_] cluster is transformed into a monomeric form with a [Fe_2_S_2_] cluster that is unable to bind to DNA (Khoroshilova et al., 1997; Zhang et al., 2012). The [Fe_4_S_4_] cluster in FNR can be regenerated from a cysteine persulfide-coordinated [Fe_2_S_2_] cluster in the presence of a reductant and ferrous iron. This observation provided some clues about a possible mechanism devoted to the repair of biological [Fe_4_S_4_] clusters, which represent the vast majority of the cellular Fe-S clusters and are usually more sensitive to oxygen compared to [Fe_2_S_2_] clusters (Zhang et al., 2012). Altogether, cluster interconversion may represent an efficient way for the maturation or repair of different types of Fe-S proteins or for the regulation of cellular processes in response either to some changes in the intracellular conditions or to extracellular stimuli.

### Biological roles of Fe-S proteins

The primary role of Fe-S proteins concerns their involvement in electron transfer reactions, usually as one-electron carriers. Hence, they are major elements for the photosynthetic electron transport chain, being present in the Rieske protein of the *b*_6_*f* complex and as three [Fe_4_S_4_] clusters at the level of photosystem I (PSI) directly transferring their electrons to stromal ferredoxins. Several Fe-S clusters also contribute to electron transfer in the respiratory electron transport chain. There are eight Fe-S clusters in complex I: two [Fe_2_S_2_] and six [Fe_4_S_4_]; three in complex II: one [Fe_2_S_2_], one [Fe_3_S_4_] and one [Fe_4_S_4_], and a Rieske protein in the *bc*_1_ complex. However, due to the chemical versatility of both iron and sulfur and the structural diversity and electronic properties of Fe-S clusters, they also have several other identified roles. The most documented functions, which will be illustrated by a few examples, are the regulation of gene expression, and the catalytic roles which include substrate binding, activation and/or reduction.

In bacteria, several transcription factors, SoxR, IscR, NsrR and FNR, possess Fe-S cofactors serving for the sensing of cellular changes in superoxide, Fe-S clusters, NO and oxygen contents, respectively (Fleischhacker and Kiley, 2011). Most of these regulatory mechanisms reflect the sensitivity of Fe-S clusters of these transcription factors to reactive species and their subsequent degradation. In eukaryotes, a well-known example is the iron regulatory protein 1 (IRP1) that controls the cellular iron homeostasis. This protein alternates between an active cytosolic aconitase holoform harboring an Fe-S cluster and an apoform that binds iron responsive elements for post-transcriptional regulation (Rouault, 2006). However, although a recombinant aconitase can bind to the 5′UTR of the *Arabidopsis* chloroplastic CuZn superoxide dismutase 2, such regulation does not seem to be a general pathway operating in plants (Arnaud et al., 2007; Moeder et al., 2007).

Besides this sensing function, Fe-S clusters also constitute the reaction center of many enzymes, serving for the binding and/or the activation of the substrate or simply for funneling electrons to the substrate. This is typically illustrated for aconitase for which one Fe atom is involved in the coordination of citrate (Kennedy et al., 1987). Another important and widespread protein family is constituted by radical-SAM enzymes that catalyze the reductive cleavage of *S*-adenosylmethionine (SAM) to generate a 5′-deoxyadenosyl radical which subsequently activates the substrate by abstracting a hydrogen atom (Atta et al., 2010). This radical chemistry is required for many biosynthesis and degradation pathways as for biotin or lipoic acid synthesis. Illustrating the importance of Fe-S clusters, it is worth mentioning the unique cluster chemistry of chloroplastic ferredoxin:thioredoxin reductase and most likely bacterial and archaeal counterparts possessing similar enzymes (Jacquot et al., 2009). Using a catalytic intermediate with two cysteinyl ligands at a unique Fe site, the active-site [Fe_4_S_4_] cluster promotes the reduction of an intramolecular disulfide bond in thioredoxins by relaying electrons provided by ferredoxins (Walters et al., 2005; Dai et al., 2007).

Finally, several recent examples of [Fe_4_S_4_] clusters bound to enzymes in DNA metabolism (glycosylases, primases, helicases, helicases/nucleases, polymerases) raised the question of the chemical role of these clusters (Wu and Brosh, 2012). However, this is currently unclear as mutation of the cluster ligands affects the integrity and functioning of some enzymes, whereas there was no measurable effect in other cases.

## The Fe-S cluster assembly machineries

**Figure 2:**
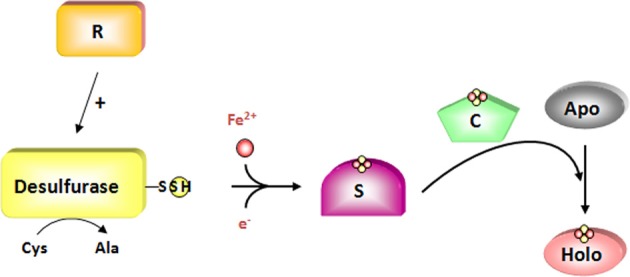
**The two-steps of *de novo* assembly of Fe-S clusters**. An Fe-S cluster is assembled on scaffold proteins (S) from iron and sulfur sulfane generated as a persulfide on cysteine desulfurase whose activity is regulated by specific proteins (R). For this step, two electrons are required to reduce sulfur sulfane (S^0^) into sulfide (S^2−^). This cluster is subsequently transferred to acceptor apoproteins *via* the action of carrier proteins (C). The cysteine desulfurase is colored in yellow and the regulators in orange. Putative scaffold and carrier proteins are colored in purple and green, respectively.

Schematically, the assembly process can be divided into two steps (Figure 2). In the first stage, Fe-S clusters are built from iron and sulfur delivered by proteins onto so-called scaffold proteins (comprising U-type scaffold proteins or a SufBCD complex). Then, the subsequent transfer of preformed Fe-S clusters to target recipient apoproteins is achieved with the help of carrier proteins (generally referred to as Nfu proteins or A-type carriers (ATC) called SufA/IscA). The nature of the iron donor is still a matter of debate, whereas sulfur is mobilized *via* the action of pyridoxal-5′-phosphate (PLP)-dependent cysteine desulfurases (SufS, IscS/Nfs) (Lill and Mühlenhoff, 2008). As sulfur is always present in the S^2−^ oxidation state in Fe-S clusters, two electrons are needed to reduce sulfane sulfur (S^0^) in the course of cluster assembly (Lill, 2009). Beyond electron donors, a few additional proteins such as ATP-hydrolyzing chaperones or sulfur acceptors are also required for some steps.

The components for Fe-S cluster assembly in eukaryotes and in particular in plants, belong to three systems, namely the SUF (sulfur mobilization), ISC (iron-sulfur cluster) and CIA (cytosolic iron-sulfur cluster assembly) machineries for plastidial, mitochondrial and cytosolic/nuclear Fe-S proteins, respectively (Lill and Mühlenhoff, 2008). In addition, an ISC export machinery links the mitochondrial and the cytosolic machineries (Lill and Mühlenhoff, 2008). Whereas the ISC export and CIA machineries are specific to eukaryotes, the SUF and/or ISC machineries are found in most living organisms. Hence, the prokaryotic systems represent good working models for the plastidial and mitochondrial assembly machineries considering the evolutionary origin of these organelles.

### The mitochondrial ISC-like system

#### Overview of the ISC system in bacteria and in mitochondria of non-plant eukaryotes

The ISC-mediated assembly of Fe-S clusters was first described in bacteria (Roche et al., 2013). In *Azotobacter vinelandii* and *E. coli*, the *isc* operon is formed by 7 genes that encode a regulator (IscR), a cysteine desulfurase (IscS), a scaffold protein (IscU), an ATC protein (IscA), a DnaK-like chaperone (HscA), a DnaJ-like co-chaperone (HscB) and an electron donor, a ferredoxin (Johnson et al., 2005). These proteins likely form transient and sequential protein complexes exhibiting a dynamic interplay of protein–protein interactions and associated conformational changes. IscS is a PLP-dependent enzyme extracting the sulfur atom from L-cysteine (Schwartz et al., 2000) and transiently binding a persulfide onto an active-site cysteine. Following the formation of an IscS-IscU complex, the sulfur atom is transferred from IscS to the scaffold protein, IscU. IscU also acts as an Fe acceptor, enabling the assembly of an Fe-S cluster, without dissociation of the IscS-IscU complex. The origin of the iron, which has been suggested to come from frataxin, named CyaY in bacteria, is still uncertain (see section Where do Iron Atoms Come From?). By interacting with IscS and/or IscU, ferredoxin provides the electrons to reduce the sulfur but it could also contribute to the dynamic equilibrium between the [Fe_2_S_2_] and [Fe_4_S_4_] cluster-bound forms of IscU by enabling the reductive coupling of two [Fe_2_S_2_]^2+^ clusters to form a single [Fe_4_S_4_]^2+^ cluster on IscU (Chandramouli et al., 2007; Kim et al., 2013). The release and transfer of the Fe-S cluster to apo-target proteins is enhanced by the interaction of IscU with HscA and HscB proteins in an ATP-dependent process. HscA and HscB belong to the DnaK/DnaJ chaperones/co-chaperones families, respectively (Hoff et al., 2000). A LPPVK sequence motif in IscU is recognized by HscA and this interaction is regulated by the co-chaperone HscB, whose interaction with IscU involves hydrophobic residues (Hoff et al., 2003; Fuzery et al., 2008). Cluster assembly and release have been shown to be uncoupled.

The cluster built on IscU is delivered to final acceptors with the help of carrier proteins which may provide the necessary specificity. Among putative carrier proteins present in *E. coli* (IscA, SufA, ErpA, NfuA, ApbC and Grx4), IscA and SufA are clearly associated with the ISC and SUF pathways, respectively, in accordance with their presence in the respective gene cluster/operon. In the current model, ErpA, which is required in particular for cluster assembly in two enzymes implicated in isoprenoid synthesis (IspG/H), is connected to both pathways, but it would act in a later step, serving as a relay of IscA or SufA. Quite similarly, NfuA is also connected to both pathways receiving an Fe-S cluster from the IscU or SufBCD scaffold proteins (Py et al., 2012). For other proteins, the situation is more uncertain. There is no clear information on the relationship of ApbC with one of these pathways. For Grx4, the fact that a mutation of individual genes of the isc operon in a *grxD*/*ydhD* recipient mutant strain leads to an aggravating phenotype, is in favor of Grx4 cooperating with the *suf* operon (Butland et al., 2008). Recently, Grx4 was found to cooperate with NfuA for the maturation of MiaB, a radical SAM-dependent enzyme involved in tRNA maturation (Boutigny et al., 2013).

The system found in eukaryotes is quite similar to the bacterial system but it is compartmentalized in mitochondria. Its integrity is required for the functioning of the CIA machinery and there are a few additional actors (Lill et al., 2012). As in bacteria, an [Fe_2_S_2_] cluster is first synthesized on the Isu1 scaffold protein, prior to its dissociation and transfer to specific ISC targeting factors responsible for its shuttling and efficient insertion into target apoproteins (Mühlenhoff et al., 2003). The *de novo* synthesis on Isu1 requires the Nfs1–Isd11 complex for providing S, the NAD(P)H-ferredoxin reductase (Arh1p) (Li et al., 2001) and ferredoxin (Yah1p in yeast) (Lange et al., 2000) for electron transfer, and possibly frataxin as an iron donor. Although purified Nfs1 can function as a cysteine desulfurase releasing sulfide *in vitro* in the presence of dithiothreitol, it has no activity in the absence of Isd11, which might have a stabilizing effect (Mühlenhoff et al., 2004; Adam et al., 2006; Wiedemann et al., 2006). Isd11 is a member of the LYR protein family and it has no ortholog in prokaryotes (Richards and van der Giezen, 2006; Atkinson et al., 2011). The dissociation of the cluster from Isu1 requires its interaction with an intermediate chaperone complex comprising Ssq1 and Jac1, the orthologous proteins to HscA and HscB. It also requires additional factors such as the nucleotide exchange factor Mge1 and the monothiol glutaredoxin Grx5 which may transiently coordinate the Fe-S cluster considering the ability of this type of Grxs to bind [Fe_2_S_2_] clusters into dimers (Rodriguez-Manzaneque et al., 2002; Mühlenhoff et al., 2003; Bandyopadhyay et al., 2008a). These steps are necessary for the maturation of all mitochondrial Fe-S proteins, but also for cytosolic and nuclear Fe-S protein biogenesis, and for transcriptional iron regulation *via* mitochondria. Therefore, these components are named “ISC core proteins.”

The maturation of certain Fe-S proteins requires additional specific proteins named ISC targeting factors, selectively interacting with subsets of Fe-S proteins (Lill et al., 2012). These late-acting components do not contribute to extra-mitochondrial processes. Isa proteins together with Iba57 are required for specific Fe-S enzymes, such as the mitochondrial aconitase and two radical SAM enzymes, the biotin and lipoic acid synthases (Gelling et al., 2008; Mühlenhoff et al., 2011; Sheftel et al., 2012). Nfu1 is required for the function of lipoic acid synthase (Navarro-Sastre et al., 2011). For organisms possessing a respiratory complex I, the P-loop NTPase Ind1 is important for its assembly (Bych et al., 2008a; Sheftel et al., 2009). Most ISC components are essential for the viability of yeast and human cells, pointing to the importance of this pathway.

#### The mitochondrial ISC system in arabidopsis thaliana

**Figure 3:**
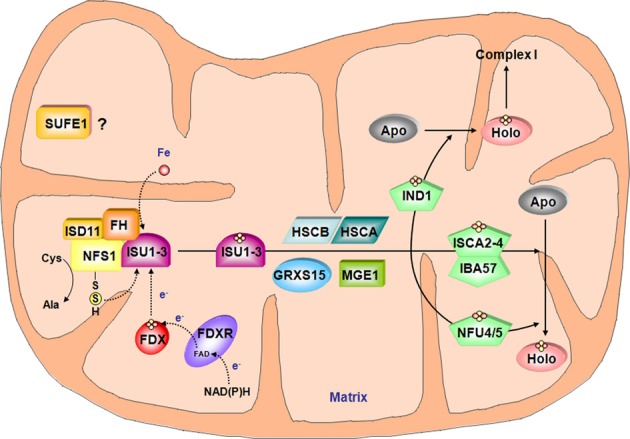
**Model for the Fe-S cluster assembly machinery in mitochondria**. This scheme has been drawn essentially based on the current models of Fe-S cluster assembly for the bacterial and the mitochondrial yeast ISC machineries. The color code is the same as in Figure 2. The complex between the cysteine desulfurase NFS1 and ISD11 mobilizes sulfur from cysteine and frataxin (FH) promotes the interaction with the scaffold protein ISUs and favor sulfur transfer reaction. In addition to iron whose origin is yet unidentified, the Fe-S cluster synthesis on ISUs also requires electrons probably coming from the ferredoxin/ferredoxin reductase system. According to the yeast model, HSCA, HSCB, MGE1 and GRXS15 may be involved in Fe-S cluster release from ISU and subsequent transfer to carrier proteins such as IND1, ISCA2-4/IBA57 couple and NFU4-5 that finally transfer the Fe-S cluster to specific target proteins. Due to its localization in mitochondria and its ability to stimulate *in vitro* the activity of NFS1, SUFE1 may also be involved in sulfur mobilization.

The basic mechanisms of the ISC system described above likely apply for plant mitochondria, as all components, both ISC core and targeting proteins, are found in *Arabidopsis* (Table 1) (Figure 3) (Balk and Pilon, 2011). The major difference is that some gene families (ISCU, ISCA and NFU) are slightly expanded compared with non-plant eukaryotes. A recombinant *Arabidopsis* cysteine desulfurase, AtNFS1, expressed and purified from *E. coli*, was able to catalyze the release of sulfide from cysteine (Frazzon et al., 2007). Furthermore, AtNFS1 promotes assembly of an Fe-S cluster *in vitro* on a recombinant AtISU1 scaffold protein in a time- and cysteine-dependent manner and it interacts with frataxin (Frazzon et al., 2007; Turowski et al., 2012). In addition to ISU1, two other ISU proteins (AtISU2 and AtISU3) are also located in *Arabidopsis* mitochondria, and all ISUs can complement a yeast Δ*isu1*Δ*nfu1* thermo-sensitive mutant strain (Leon et al., 2005). It is not yet clear which interacting protein is required for AtNFS1 function since the plant ISD11 ortholog has not been functionally characterized and an additional interacting protein of AtNFS1, AtSUFE1, is found in mitochondria (Xu and Moller, 2006). Although the majority of an overexpressed SUFE1-GFP fusion was found to be localized to plastids, contribution of AtSUFE1 for the mitochondrial ISC machinery is supported by the observation that a plastidial targeted SUFE1 is not sufficient for complementing the embryo lethality of the mutant (Xu and Moller, 2006; Ye et al., 2006a).

**Table 1:** ***Arabidopsis thaliana* members of the mitochondrial ISC machinery**.

**Protein names**	**AGI numbers**	**Phenotype(s) of mutant plants**	**Reference(s)**
NFS1	At5g65720	Embryo lethal	Frazzon et al., 2007
SUFE1	At4g26500	Embryo lethal	Xu and Moller, 2006
ISD11	At5g61220	None yet described	
ISU1	At4g22220	Likely embryo-lethal but only knock down by RNAi was achieved	Frazzon et al., 2007
ISU2	At3g01020	None yet described	
ISU3	At4g04080	None yet described	
FH	At4g03240	Embryo lethal	Busi et al., 2006
mFDX1	At4g05450	None yet described	
mFDX2	At4g21090	None yet described	
mFDXR	At432360	None yet described	
HSCA1	At4g37910	None yet described	
HSCA2	At5g09590	None yet described	
HSCB	At5g06410	Reduced seed set, waxless phenotype, inappropriate trichome development, decreased Fe-S enzyme activities	Xu et al., 2009
MGE1a	At4g26780	None yet described	
MGE1b	At5g55200	None yet described	
ISCA2	At2g16710	None yet described	
ISCA3	At2g36260	None yet described	
ISCA4	At5g03905	None yet described	
NFU4	At3g20970	None yet described	
NFU5	At1g51390	None yet described	
IND1-like/INDH	At4g19540	None yet described	
IBA57.1	At4g12130	Embryo lethal	Waller et al., 2012
GRXS15	At3g15660	Sensitivity to H_2_O_2_	Cheng, 2008

*A. thaliana SUFE1 is dual targeted to plastids and mitochondria, AtHSCA and AtHSCB to the mitochondria and the cytosol. For ATCs (ISCA2 to ISCA4), the mitochondrial localization is based only on prediction and has not been experimentally confirmed*.

As reported above, dissociation of the Fe-S cluster from the Nfs1-Isu1 complex requires accessory proteins in yeast mitochondria.* A. thaliana* HSCB, the yeast Jac1 ortholog, was shown to interact with the scaffold AtISU1 by yeast-two-hybrid and bimolecular fluorescence complementation (BiFc) (Xu et al., 2009). Moreover, it is able to complement a yeast *jac1* mutant strain. One of two abundant mitochondrial HSP70 chaperones, HSCA1 protein, has its ATPase activity stimulated *in vitro* by HSCB. Nevertheless, its involvement in Fe-S cluster assembly in mitochondria has not been investigated yet (Heazlewood et al., 2004). The involvement of the mitochondrial GRXS15 in the process of dissociation remains to be confirmed as this is the only plant monothiol Grx unable to complement the yeast *grx5* mutant (Bandyopadhyay et al., 2008a).

Among specialized targeting factors, very scarce functional information is available, although orthologs are present in *Arabidopsis* (Table 1). For NFU members, the mitochondrial localization has only been confirmed for NFU4 (Leon et al., 2003). NFU5 has, however, been identified in two proteomic studies performed with isolated mitochondria (Ito et al., 2006; Tan et al., 2010). From a functional point of view, both *A. thaliana* NFU4 and NFU5 are able to complement the yeast Δ*isu1*Δ*nfu1* thermo-sensitive mutant strain (Leon et al., 2003). Whether they participate to the general assembly pathway or to cluster assembly in specific Fe-S proteins awaits confirmation. The *Arabidopsis* mitochondrial IBA57 is an essential protein and seems to have conserved functions compared to other organisms. In particular, it is able to complement an *E. coli* mutant strain for the ygfZ ortholog by rescuing MiaB activity (Waller et al., 2010, 2012). *A. thaliana* IND1/INDH was found in mitochondria and it is expected, by analogy with other eukaryotes that it plays a role in the maturation of Fe-S proteins in complex I (Bych et al., 2008b). Finally, there is no information about the roles, subcellular localizations and properties of the three putative ATC isoforms (ISCA2 to 4) predicted to be targeted in mitochondria.

### The chloroplastic SUF-like system

#### Overview of the SUF system in bacteria

Based on the organization of the *suf* operon in *E. coli*, the SUF system is primarily composed of six proteins. However, the situation is more complex as some bacteria have different operon architectures and other biogenesis factors encoded by isolated genes (Py et al., 2011). From genetic and biochemical investigations performed in *E. coli*, the current view is that SufE and SufS are involved in sulfur mobilization from cysteine. SufB, C and D form a complex where SufB harbors both the *de novo* assembled Fe-S cluster and a flavin redox cofactor, which could transmit the electrons required for reducing the sulfur. The ATC protein SufA likely acts as a carrier protein, but this assumption is challenged by the fact that SufA and ATCs in general can also bind mononuclear iron, sometimes with a good affinity (Fontecave et al., 2005; Lu et al., 2008; Chahal et al., 2009; Gupta et al., 2009; Wollers et al., 2010). A recent study added a higher level of complexity by showing that SufBC_(2)_D and SufB_(2)_C_(2)_ complexes harboring a preformed [Fe_4_S_4_] cluster can serve for the maturation of [Fe_2_S_2_]-containing SufA or ferredoxin (Chahal and Outten, 2012), but also of NfuA (Py et al., 2012). These results support the view that SufBCD complexes, irrespective of their detailed composition, constitute the scaffold system assembling nascent Fe-S clusters which can be loaded on SufA or NfuA carriers for *in vitro* maturation of [Fe_2_S_2_] enzymes like Fdx. As mentioned earlier, other proteins, not belonging to the *suf* operon, have been functionally associated with some components of the SUF machinery in *E. coli*. For instance, the csdA-csdE couple constitutes an additional cysteine desulfurase-sulfurtransferase system likely fueling the SufBCD complex (Loiseau et al., 2005; Trotter et al., 2009).

In cyanobacteria, Fe-S cluster biogenesis relies both on the ISC and SUF systems but only the latter is essential (Balasubramanian et al., 2006). Important differences with *E. coli* include the presence of a SufR repressor, of four cysteine desulfurases (two IscS, one SufS and one CsdA) and the absence of an IscU prototype. While a *sufA iscA* mutant in *E. coli* is not viable under aerobiosis (Vinella et al., 2009), a similar double mutant in *Synechococcus* sp PCC 7002 has similar growth to the wild type both under standard and stress conditions (Balasubramanian et al., 2006). The most notable difference is the accumulation of transcripts for some Isc and Suf components in response to iron limitation and to oxidative stress which suggested that SufA and IscA may play regulatory roles in iron and/or redox sensing (Balasubramanian et al., 2006). On the contrary, inactivation of Nfu in this species is lethal pointing to an essential function likely related to PSI assembly as shown by *in vitro* experiments (Balasubramanian et al., 2006; Jin et al., 2008).

#### The chloroplastic SUF system in arabidopsis thaliana

**Figure 4:**
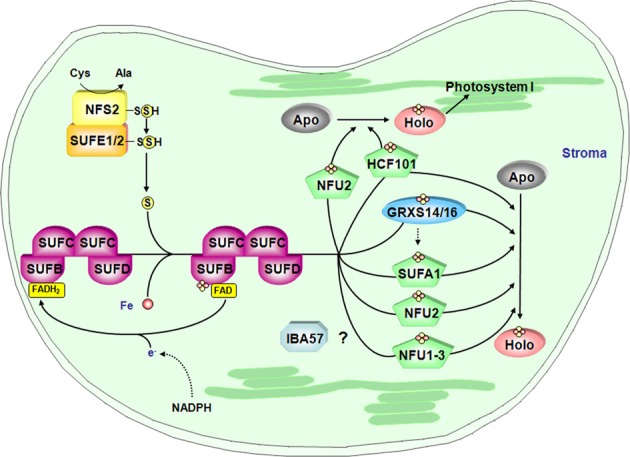
**Model for the Fe-S cluster assembly machinery in chloroplasts**. This scheme has been drawn essentially based on the current models of Fe-S cluster assembly for the plant plastidial and bacterial SUF assembly machineries. The color code is the same as in Figure 2. SUFE1/2 stimulate the cysteine desulfurase activity of NFS2 and transfer the sulfur to SUFB that fulfills scaffold protein function by forming a complex with SUFC and SUFD. The iron source is unknown and electrons may be channeled from NADPH to a SUFB-bound FAD *via* an as yet unidentified flavin reductase. Fe-S cluster transfer to specific proteins is then accomplished by carrier proteins. Hence, NFU2 and HCF101 are involved in the maturation of one or several proteins belonging to PSI and some other stromal proteins. Up to now, target proteins of SUFA1, NFU1 and 3 are not identified. Finally, some plants possess a plastidial isoform of IBA57. By analogy with the mitochondrial isoform, plastidial IBA57 might act as carrier protein in conjunction with SUFA1 but this assumption awaits confirmation. Finally, the role of GRXS14 and GRXS16 is uncertain but they may be involved in Fe-S cluster release from scaffold protein as in mitochondria and/or they could be carrier proteins for the delivery of Fe-S clusters to specific target proteins.

A SUF-like system exists in photosynthetic organisms: cyanobacteria, algae and terrestrial plants (Figure 4). Some additional assembly proteins have been identified in *A. thaliana* compared to the *E. coli* SUF machinery (Table 2). Among the core components, NFS2, SUFB, SUFC and SUFD are encoded by a single gene. There are three plastidial proteins containing a SufE domain. Considering that it is expressed in most tissues, SUFE1 might be the preferential activator of NFS2 in plastids (Ye et al., 2006a). It is worth noting that SUFE1 is unique to plants, having a C-terminal BolA domain that could contribute to its post-translational regulation (see section Post-Translational Control: Does the Grx/BolA Interaction also Constitute a Regulatory Link between Fe-S cluster Biogenesis and Cellular Iron Regulation in Plants?). The other two genes, *SUFE2* and *SUFE3*, have respectively, a specific expression in flowers and a low transcript level in all major plant organs (Murthy et al., 2007). All three SUFE proteins can stimulate NFS2 activity *in vitro*, and SUFE3 also possesses quinolinate synthase activity owing to the presence of a C-terminal NadA domain. No phenotype has yet been described for *sufE2* knock-out (KO) mutant, but the disruption of *SUFE1* and *SUFE3* genes results in arrested embryo development (Xu and Moller, 2006; Murthy et al., 2007). Basically, all KO mutants for these early-acting core components described thus far are embryo-lethal (Table 2) (Xu and Moller, 2004, 2006; Hjorth et al., 2005; Murthy et al., 2007; Van Hoewyk et al., 2007; Nagane et al., 2010).

**Table 2:** ***Arabidopsis thaliana* members of the plastidial assembly machinery**.

**Protein names**	**AGI numbers**	**Phenotype(s) of mutant plants**	**Reference(s)**
NFS2/CpNifS	At1g08490	A RNAi mutant is seedling lethal	Van Hoewyk et al., 2007
SUFE1	At4g26500	Embryo lethal	Xu and Moller, 2006
SUFE2	At1g67810	None yet described	
SUFE3	At5g50210	Embryo lethal	Murthy et al., 2007
SUFB/ NAP1	At4g04770	Strong alleles are embryo-lethal, weak alleles present a perturbation in the phytochrome signal transduction pathway and an impaired chlorophyll degradation	Moller et al., 2001; Nagane et al., 2010
SUFC/ NAP7	At3g10670	Embryo lethal	Xu and Moller, 2004
SUFD/ NAP6	At1g32500	Bleached plants with reduced root growth, defects in plastid morphology and in seed germination	Hjorth et al., 2005
SUFA1/CpISCA	At1g10500	No phenotype	Yabe and Nakai, 2006
NFU1	At4g01940	None yet described	
NFU2	At5g49940	Pale green, dwarf phenotype, inhibition of root growth. Impaired PSI and decreased levels of FDX	Touraine et al., 2004; Yabe et al., 2004
NFU3	At4g25910	None yet described	
HCF101	At3g24430	Strong alleles are seedling-lethal In weak alleles, reduced levels of [4Fe-4S] enzymes (PSI, FTR)	Lezhneva et al., 2004
IBA57.2	At1g60990	None yet described	
GRXS14	At3g54900	Increased carbonylation of plastidial proteins	Cheng et al., 2006
GRXS16	At2g38270	None yet described	

*The plastidial localization of all these proteins has been experimentally confirmed. A. thaliana SUFE1 is dual targeted to plastids and mitochondria. There is another putative SUFB gene (At4g04770) but, from EST or transcriptomic data, there is no evidence that it is expressed*.

On the contrary, plant KO mutants for most genes coding targeting factors (generally carrier proteins) acting late during Fe-S cluster biogenesis, are viable. This may indicate either that there is redundancy among them or that their function is dispensable i.e., the transfer of preformed clusters from scaffold proteins to acceptor proteins could eventually occur *in vivo* in the absence of carrier proteins, though maybe at lower rates or lower specificities. To date, the only exception is an *hcf101* mutant, whose lethal phenotype might be explained by the essential nature of its targets, i.e., one or several of the three Fe-S proteins of PSI (Lezhneva et al., 2004; Stockel and Oelmuller, 2004). Other proteins have been proposed, based on *in vitro* or *in vivo* data, to participate in the assembly of Fe-S proteins in chloroplasts. For instance, the possible functioning of Grxs as carrier proteins was supported by data showing that two plant chloroplastic Grxs (GRXS14 and S16), which bridge one [Fe_2_S_2_] cluster per homodimer, can rapidly and stoichiometrically transfer their cluster to an apo Fdx and that they complement a yeast *grx5* mutant (Bandyopadhyay et al., 2008a). Among the three plastidial NFU proteins, NFU1 to NFU3, NFU2 is the best functionally characterized. The domain organization of the plastidial NFU proteins is specific to photosynthetic eukaryote organisms. Nevertheless, both NFU1 and NFU2 are able to restore the growth of an *isu1 nfu1* yeast mutant, when targeted to yeast mitochondria (Leon et al., 2003). The phenotypic and physiological analysis of *nfu2* KO plants indicated that NFU2 contributes to the correct assembly of leaf FDX and PSI (Touraine et al., 2004; Yabe et al., 2004). Concerning ATC proteins, a KO mutant for *AtSUFA1*/*CpISCA* has no visible phenotype, although transcripts are found in most tissues and particularly in leaves (Yabe and Nakai, 2006). Nevertheless, biochemical evidence supported a role of SUFA1 in the SUF machinery. AtSUFA1 is able to transiently bind an [Fe_2_S_2_] upon *in vitro* reconstitution that can be transferred to an apo-ferredoxin (Abdel-Ghany et al., 2005). On the other hand, the observation of a rapid, unidirectional and intact transfer of an [Fe_2_S_2_] cluster from *A. thaliana* GRXS14 suggested possible physiological sequential steps for Fe-S cluster shuttling with GRXS14 acting before SUFA1 (Mapolelo et al., 2013).

### The cytosolic CIA system

The CIA machinery is required for the maturation of cytosolic and nuclear Fe-S proteins and is restricted to eukaryotes. Owing to the absence of a cytosolic cysteine desulfurase, its functioning depends on the mitochondrial ISC machinery. Indeed, it was shown in yeast and human that mitochondrial Nfs1 is required for the assembly of extra-mitochondrial Fe-S proteins (Mühlenhoff et al., 2004; Kispal et al., 2005; Biederbick et al., 2006). Its fundamental role has been emphasized in recent years by the identification of several Fe-S proteins involved in DNA/RNA metabolism (White and Dillingham, 2012; Wu and Brosh, 2012). Whereas most of the CIA components have been first identified in yeast and mammals, orthologs are also present in plants (Table 3).

**Table 3:** ***Arabidopsis thaliana* members of the CIA and ISC export machineries**.

**Protein names**	**AGI numbers**	**Phenotype(s) of mutant plants**	**Reference(s)**
NAR1/GOLLUM	At4g16440	Embryo lethal	Cavazza et al., 2008; Luo et al., 2012
CIA1	At2g26060	Embryo lethal	Luo et al., 2012
NBP35	At5g50960	Embryo lethal	Bych et al., 2008b
AE7/ MIP18/CIA2	At1g68310	Strong allele is embryo lethal, weak alleles are viable but exhibit highly accumulated DNA damage and cell cycle arrest	Yuan et al., 2010; Luo et al., 2012
MET18/MMS19	At5g48120	No phenotype under standard growth conditions	Luo et al., 2012
DRE2/CIAPIN	At5g18400	Embryo lethal	Bernard et al., 2013
TAH18/ATR3	At3g02280	Embryo lethal	Varadarajan et al., 2010
ATM3/ABCB7	At5g58270	Defects in root growth, chlorophyll content and seedling establishment	Kushnir et al., 2001; Kim et al., 2006; Bernard et al., 2009
ERV1/ALR	At1g49880	Embryo lethal	Carrie et al., 2010

*Except ATM3 and ERV1 which code for the two protein components of the ISC machinery, all other genes code for proteins of the CIA machinery. Yeast and human names have been indicated. ATM1 (At4g28630), ATM2 (At4g28620), AE7.2/AEL1 (At3g50845) and AE7.3/AEL2 (At3g09380) genes have not been listed here considering that they are not able to complement atm3 and ae7 mutants. There are putative additional CIA1 (At4g32990) and DRE2 genes (At5g18362) but, from EST or transcriptomic data, there is no evidence that they are expressed*.

#### The CIA machinery in yeast and mammals

As already described for ISC and SUF assembly machineries, the first step of the CIA machinery consists of the *de novo* assembly of an Fe-S cluster onto scaffold proteins using three elements, sulfur, iron and electrons. To date, the source of iron is unknown. The sulfur is provided by the mitochondrial ISC machinery in the form of an unidentified sulfur-containing compound that is transported through the membranes (see section The ISC Export Machinery). The required electrons are initially delivered from NADPH and they are channeled through the FAD and FMN cofactors of the NADPH-dependent diflavin reductase Tah18 to the Fe-S protein Dre2 (Zhang et al., 2008; Netz et al., 2010; Banci et al., 2013). Interestingly, the maturation of Dre2 is dependent on the ISC but not on the CIA machinery indicating either that this is the primary Fe-S scaffold or that there is another pathway for the assembly of Fe-S clusters into this protein. However, the fact that it is unable to transfer its Fe-S cluster to target proteins as apo-ferredoxin (Yah1) and apo-isopropylmalate isomerase (Leu1) precluded considering Dre2 as a scaffold protein (Netz et al., 2010). Surprisingly, whereas Tah18 and Dre2 are required for Fe-S cluster assembly on both Nbp35 and Nar1, they are not for Cfd1.

In yeast and human, the scaffold function is ensured by two P-loop NTPases, Nbp35 and Cfd1, which form a tight heterotetrameric complex ligating four [Fe_4_S_4_] clusters, one at the C-terminus of each protein and two permanent clusters situated in the N-terminal region of the two Nbp35 monomers (Netz et al., 2007, 2012). The transfer of the Fe-S cluster to final acceptor proteins requires additional participants. The maturation of one of them, Nar1, is intriguing since it is both a target and a component of the CIA machinery. Indeed, the biochemical characterization of the yeast protein which exhibits a strong homology to [FeFe] hydrogenases revealed that it can bind two [Fe_4_S_4_] clusters, one which seems to be permanently present and one which is transferred to recipient proteins (Balk et al., 2004; Urzica et al., 2009). The Cia1 protein, which possesses a WD40 repeat domain, likely facilitates the transfer of the Fe-S cluster to acceptor proteins by interacting with Nar1 (Balk et al., 2005). More recently, two additional components, MET18/MMS19 and MIP18 (MMS19-interacting protein) were shown to bind to the Nar1/Cia1 complex in order to shuttle Fe-S clusters to a specific subset of Fe-S proteins, especially those involved in DNA metabolism (Weerapana et al., 2010; Gari et al., 2012; Stehling et al., 2012).

This quite simple model might well be more complex in mammalian cells considering the identification of a small cytosolic pool of ISC proteins, ISCS and ISCU1, in cultured human cells (Biederbick et al., 2006; Tong and Rouault, 2006). However, there is no clear evidence for their *in vivo* requirement for Fe-S assembly in this compartment. Besides, some other proteins could belong to the CIA machinery. In yeast, depletion of cytosolic Grx3 and Grx4 clearly affects cytosolic and nuclear Fe-S biogenesis but it also leads to defects in both the mitochondrial ISC machinery and the synthesis of heme and di-iron centers (Mühlenhoff et al., 2010). It has led to the proposal that these Grxs might function both in iron sensing and in intracellular iron delivery. Interestingly, human Dre2/anamorsin/Ciapin-1 was found to interact with the human Grx3 ortholog called PICOT (PKC-interacting cousin of thioredoxin) by yeast two hybrid, confirming previous high throughput data obtained in yeast (Tarassov et al., 2008; Saito et al., 2011). This interaction may also support the observation that Grx3/4 and Dre2 serve for the assembly of the di-ferric Tyr^•^ cofactor in yeast ribonucleotide reductase (Zhang et al., 2011). Overall, based on the strict definition that CIA components are only required for extra-mitochondrial Fe-S biogenesis, it seems that cytosolic monothiol Grxs cannot be considered as a CIA component *per se*. However, (i) the assembly of the Fe-S cluster in yeast Grx3/4 is not dependent on the CIA, similar to Cfd1 and Dre2, (ii) the depletion of Grx3 and 4 in the W303 genetic background is lethal and (iii) Fe-S clusters ligated by glutathione may be physiologically relevant (Mühlenhoff et al., 2010; Qi et al., 2012). These observations put therefore these Grxs at a central position, possibly for the primary building of an Fe-S cluster using the mitochondrial exported sulfur compound, and/or for the subsequent delivery of Fe or Fe-S centers.

#### The CIA machinery in arabidopsis thaliana

**Figure 5:**
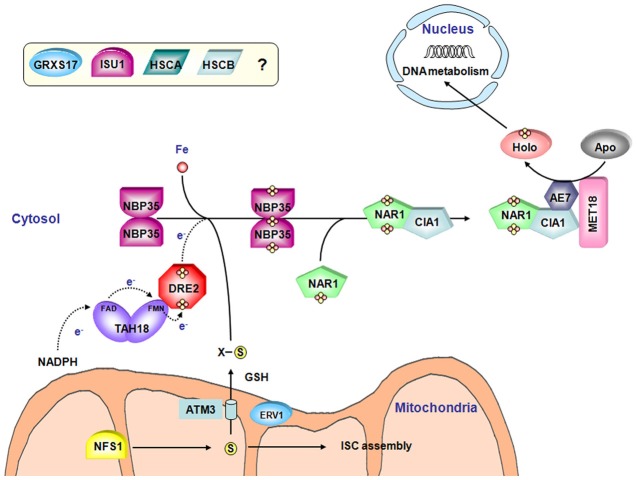
**Model for the Fe-S cluster assembly machinery in the cytosol**. Both the CIA machinery and the connected ISC export machinery have been represented. The color code is the same as in Figure 2. A sulfide compound originating from NFS1 activity and preferentially transported by the ATM3 transporter may represent the sulfur source for Fe-S cluster biogenesis in the cytosol. ERV1, another mitochondrial protein and glutathione are also important for this process although their specific roles are not elucidated. As for organellar assembly machineries, the iron source is also unclear. Based on yeast and human models of the CIA machinery assembly, TAH18 transfers electrons from NADPH to DRE2. In plants, NBP35 constitutes the sole scaffold protein. Then, the Fe-S cluster is transferred to target apoproteins *via* a NAR1-CIA1-AE7-MET18 complex. The involvement of GRXS17 and of ISU1, HSCA and HSCB in the cytosolic Fe-S cluster biogenesis is not yet elucidated.

The CIA machinery in plants should in principle be very similar to the one existing in other eukaryotic organisms, as plants possess orthologs of all identified CIA components except Cfd1 (Figure 5) (Table 3). Interestingly, except MMS19, all characterized genes are essential. The corresponding KO mutants are embryo-lethal highlighting the importance of one or several cytosolic or nuclear Fe-S enzymes (Bych et al., 2008b; Varadarajan et al., 2010; Luo et al., 2012; Bernard et al., 2013). They are mostly encoded by single genes which are usually constitutively expressed *in planta*.

As in other eukaryotes, the electrons required for Fe-S cluster assembly are probably relayed by a TAH18-DRE2 complex. For instance, the only partner of TAH18 also called ATR3 identified using a yeast 2-hybrid screen is DRE2 (Varadarajan et al., 2010). Interestingly, *A. thaliana* DRE2 can complement the yeast *dre2* mutant strain only upon AtTAH18 co-expression suggesting that it cannot interact with the yeast Tah18 ortholog (Bernard et al., 2013).

In the absence of Cfd1, NBP35 fulfils the scaffold function alone (Bych et al., 2008b; Kohbushi et al., 2009). The *A. thaliana* isoform does not complement a yeast *nbp35* mutant strain, whereas it does partially complement the yeast *cfd1* mutant strain suggesting that it does not have the capacity to interact with yeast Cfd1. The biochemical characterization of AtNBP35 revealed that it is a dimer able to bind a stable [Fe_4_S_4_] cluster at the N-terminal part of each monomer and a more labile C-terminal [Fe_4_S_4_] cluster bridged by the two monomers and which can be transferred *in vitro* to a yeast apo-Leu1 (Bych et al., 2008b).

The role of the targeting factors (NAR1, CIA1, AE7/MIP18 and MET18/MMS19) has been investigated recently in plants. The cytosolic aconitase and the nuclear DNA glycosylase ROS1, both containing [Fe_4_S_4_] clusters, have decreased activities in a weak *ae7* allele, whereas the [Fe_2_S_2_]-containing aldehyde oxidase is unaffected by this mutation (Luo et al., 2012). It suggested that AE7 might be involved preferentially in [Fe_4_S_4_] cluster maturation. The observed defects in the maintenance of nuclear genome integrity are likely associated with the Fe-S cluster assembly defect in various Fe-S proteins involved in DNA metabolism such as ROS1 (Luo et al., 2012). Using several complementary approaches (yeast two-hybrid, co-immunoprecipitation and BiFc) it was shown that AE7 interacts with both CIA1 and MET18 but not with NAR1, which led to a model slightly different from the one proposed in mammals (Figure 5). In contrast to other partners forming this complex in plants and to *mms19* mutant in human, an *A. thaliana met18* KO mutant is viable and does not exhibit obvious phenotype compared to wild type plants. However, there is a genetic interaction between *AE7* and *MMS19/MET18* because a double mutant is non-viable (Luo et al., 2012). It raises therefore the question of the role of MMS19 in plants. AE7 can either bypass the effect of a MMS19 depletion by interacting with target proteins. Alternatively, proteins targeted by MMS19 are slightly different and do not have vital functions.

A role for the plant ortholog of GRX3/PICOT, named GRXS17, is not yet elucidated. The plant proteins are slightly different from the yeast and human counterparts having one and two additional Grx domains, respectively, being composed of an N-terminal Trx-like domain followed by three successive Grx domains (Couturier et al., 2009). To date, it has been demonstrated that a *grxS17* mutant is hypersensitive to high temperature conditions (Cheng et al., 2011). The authors suggested that GRXS17 might participate to the regulation of redox homeostasis and auxin perception in the temperature-dependent post-embryonic growth but a clear link to Fe-S cluster biogenesis is missing.

Finally, a puzzling piece of data is the observation, using overexpressed fusion proteins with YFP that AtHSCB together with AtHSCA1 and AtISCU1 may also be localized in the cytosol (Xu et al., 2009). Whether they play a role in the biogenesis of cytosol and nuclear Fe-S proteins requires further experimental support, as cleavage of such translational fusions may occur.

## Inter-organellar transport and signaling

### The ISC export machinery

The ISC export machinery connects the ISC to the CIA assembly machineries and has been characterized initially in yeast by showing that a deficiency in the mitochondrial Nfs1 and in the Atm1 ABC transporter affected the maturation of cytosolic Fe-S proteins (Figure 5) (Kispal et al., 1999). In eukaryote photosynthetic organisms, the SUF machinery and in particular NFS2 is not involved in the function of the CIA or ISC machineries (Van Hoewyk et al., 2007; Bernard et al., 2013). As the components of the ISC export machinery seem to be conserved in all eukaryotes, this system will be discussed in a single part.

As mentioned earlier, the mutation of any single ISC core components in yeast led to a deficiency in the biosynthesis of cytosolic and nuclear Fe-S proteins. Besides, it was shown that similar physiological and cellular defects were observed by (i) deleting or disrupting a mitochondrial ATP-binding cassette transporter, Atm1 in yeast, ABCB7 in human and ATM3 (STA1/ABCB25) in *A. thaliana* (Kispal et al., 1999; Kushnir et al., 2001; Bernard et al., 2009), (ii) deleting the sulfhydryl oxidase Erv1 (Lange et al., 2001) and (iii) decreasing GSH levels (Sipos et al., 2002). In yeast, a depletion in *atm1* or in the gene encoding the first GSH biosynthesis enzyme, *gsh1*, leads to the accumulation of iron in the mitochondrial matrix (Sipos et al., 2002; Kispal et al., 2005). In these mutants, only the maturation of extra-mitochondrial Fe-S proteins was affected. A double *gsh1atm1* mutant is non-viable (Sipos et al., 2002).

There are two other proteins related to ATM3 in *A. thaliana*, ATM1 and ATM2, but none is involved in this process, being unable to complement the yeast mutant (Chen et al., 2007). The *atm3 Arabidopsis* plants are dwarfed and chlorotic, and present defects in root growth, chlorophyll content, seedling establishment and genome integrity (Kushnir et al., 2001; Bernard et al., 2009; Luo et al., 2012). Interestingly, although *Arabidopsis* ATM3 can functionally complement the yeast *atm1* mutant, iron accumulation in mitochondria and iron homeostasis defects in *atm3* mutant plants were not observed (Chen et al., 2007; Bernard et al., 2009). This difference may indicate that the signal produced by the ISC machinery and exported from the mitochondria, though unknown, could be a sulfide compound rather than an Fe-S cluster form. Furthermore, mutants in *ATM3* accumulate cyclic pyranopterin monophosphate, the first intermediate of Moco biosynthesis, and have decreased amounts of Moco (Kim et al., 2006; Teschner et al., 2010). More generally, it is puzzling that yeast *atm1* or *Arabidopsis atm3* KO mutants are viable, whereas there are many essential Fe-S proteins in the cytosol and nucleus, and that mutants for all core CIA genes are lethal. It would suggest that the exported mitochondrial compound is either able to partially diffuse across membranes or that it can be transported by other transporter(s) though with lower efficiency.

Erv1 constitutes one of the principal component of the oxidative protein folding in the intermembrane mitochondrial space (IMS), together with Mia40 (Herrmann and Riemer, 2010). In yeast, *erv1* gene is essential for cell viability and for the biogenesis of functional mitochondria (Lisowsky, 1994). In *A. thaliana*, the disruption of the gene is also lethal (Carrie et al., 2010). Using a conditional *erv1* yeast mutant, Erv1 was shown to be involved in cell division, in the maintenance of mitochondrial genomes and in Fe-S cluster biogenesis (Lisowsky, 1994; Lange et al., 2001). The homologous mammalian protein ALR (augmenter of liver regeneration) is able to complement the yeast mutant indicating they have a conserved function (Lange et al., 2001). Whether the contribution of Erv1/ALR is direct *via* its oxidoreductase activity or an as yet unidentified function, or indirect by ensuring the correct folding of another contributor is not known. The demonstration that GSH is involved in this process would be in favor of a redox mechanism. This is in line with microarray analyses performed in yeast presenting low GSH levels which clearly indicates that one of the primary function of GSH is related to the regulation of iron homeostasis (Kumar et al., 2011). Hence, the redox buffering function described for plants for example may not be as important in yeast, since depletion of the GSH pool in yeast does not dramatically affect the survival of the strain until a certain limit. It was recently observed that the glutathione redox potential in the IMS impacts Mia40 redox state (Kojer et al., 2012) but, on the other hand that Mia40 could bind an Fe-S cluster *in vitro* and possibly *in vivo* (Daithankar et al., 2009). However, the lethal deletion of Mia40 in yeast has not been associated to defects in the maturation of Fe-S proteins and *Arabidopsis mia40* mutant plants have no visible phenotype, although they display a decreased amount of complex I (Chacinska et al., 2004; Carrie et al., 2010). This suggests that the phenotype observed following depletion of Erv1 may not be related to a defect in the folding of a protein required for Fe-S cluster assembly and likely relies on a Mia40-independent function.

### Membrane-anchored neet proteins

MitoNEET is an Fe-S protein present in most living organisms except fungi. It was first identified as a target of piogliazone, an anti-diabetes drug (Colca et al., 2004). It is an outer mitochondrial membrane-anchored protein with one CDGSH domain oriented toward the cytoplasm (Wiley et al., 2007a). The CDGSH domain refers to a 39 amino acid motif [C-X-C-X2-(S/T)-X3-P-X-C-D-G-(S/A/T)-H] which contains the residues involved in Fe-S cluster binding. The mitoNEET proteins are dimers containing one [Fe_2_S_2_] cluster bound to each monomer and coordinated by 3 Cys and 1 His (Lin et al., 2007; Paddock et al., 2007; Wiley et al., 2007b; Conlan et al., 2009; Nechushtai et al., 2012). Substitution of the His by a Cys stabilizes the cluster and prevents its transfer to acceptor protein, revealing that this atypical coordination involving a single His residue is responsible for the relative instability of the cluster and the ability of mitoNEET to transfer it (Tirrell et al., 2009; Dicus et al., 2010; Conlan et al., 2011; Zuris et al., 2011). Moreover, only an oxidized mitoNEET can transfer its Fe-S cluster to an acceptor protein, revealing that the oxidation state of mitoNEET influences its transfer ability. Hence, it was hypothesized that changes in the cytosolic redox potential, which is normally a reducing environment precluding Fe-S transfer, may favor the transfer of the Fe-S cluster associated to the oxidized form of mitoNEET (Zuris et al., 2011). In agreement with these results, physiological concentrations of NADPH could destabilize the mitoNEET Fe-S cluster and regulate both the cellular level of holo mitoNEET, and/or its ability to transfer its cluster (Zhou et al., 2010; Zuris et al., 2012). Altogether these data, coupled to the existence of fusion proteins composed of a CDGSH domain and a FMN-binding domain in some prokaryotes, support the view of an involvement of mitoNEET protein in redox chemistry either in electron or in Fe-S cluster transfer.

Despite this, the physiological function of mitoNEET remains unclear. It could be linked to mitochondrial and/or cytosolic maturation of Fe-S clusters owing to its capacity to transfer Fe-S clusters (Paddock et al., 2007; Zuris et al., 2011; Nechushtai et al., 2012). More generally the analysis of *mitoNEET*-null mutant mice revealed that mitoNEET modulates the mitochondrial respiratory capacity possibly by controlling the iron content. Indeed, the reduction in mitoNEET expression in adipocytes enhanced both the iron content in the matrix and oxidative stress (Wiley et al., 2007a; Kusminski et al., 2012). In plants, there is a single gene that encodes a protein that is dual targeted to both the chloroplast and mitochondria (Nechushtai et al., 2012). While there is no *A. thaliana* KO plants for AtNEET available (this gene is likely essential), knockdown mutants displayed late greening and early senescence phenotypes and a reduced growth on low Fe level, but on the other hand, plants are insensitive to high Fe levels (Nechushtai et al., 2012). In addition, these plants accumulated higher levels of ROS and iron. Hence, all these results suggested that NEET would participate in Fe or Fe-S distribution between the different sub-cellular compartments, but further experiments to investigate the effect on the Fe-S cluster assembly are necessary.

## Evolution of [Fe-S] biogenesis systems in photosynthetic organisms

Considering the importance of Fe-S proteins for plant physiology and development, we sought to analyze the gene content for these assembly factors in various organisms along the green lineage to understand how gene families are conserved and potential novelties that appeared during evolution. The analysis of several sequenced genomes of photosynthetic organisms from cyanobacteria to higher plants revealed that the proteins are usually encoded by a relatively constant number of genes in all organisms and that core proteins of each machinery are mostly encoded by a single gene copy. However, some gene families have been specifically expanded in a few organisms. Another interesting evolutionary feature of these systems is the appearance of multidomain proteins in some species and the presence of similar protein domains in different assembly factors. Some novelties are detailed below.

**Figure 6:**
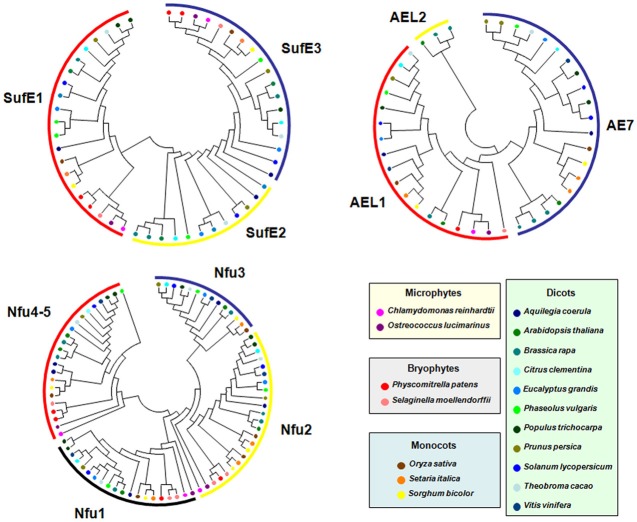
**Evolution of selected Fe-S biogenesis components in photosynthetic organisms**. Proteins belonging to the SUFE, AE7 and NFU families have been retrieved in 17 other genomes from microphytes, bryophytes, monocots and dicots available in the Phytozome (version 9.1) database (http://www.phytozome.net/) by BLASTP or TBLASTN using Arabidopsis amino acid sequences. The amino acid sequence alignments were done using CLUSTALW and imported into the Molecular Evolutionary Genetics Analysis (MEGA) package version 4.1 for the phylogenetic analysis. Phylogenetic analyses were conducted using the neighbor-joining (NJ) method implemented in MEGA, with the pairwise deletion option for handling alignment gaps, and with the Poisson correction model for distance computation. Bootstrap tests were conducted using 1000 replicates. Branch lengths are proportional to phylogenetic distances. For more clarity, protein names have been removed and replaced by colored circles corresponding to specific organisms.

Among CIA components, the case of MIP18/AE7 family is intriguing. Indeed, there are generally 1–3 genes coding for AE7-related proteins in all organisms analyzed. When 3 genes are present, they clearly form three separate clades. In *A. thaliana*, which possesses three genes, the phenotypic defects of the *ae7* mutant cannot be rescued by the two paralogs, *AEL1* and *AEL2* (Luo et al., 2012). Interestingly, the only gene present in algae, in the moss *Physcomitrella patens* and in the pteridophyte *Selaginella moellendorffii*, supposed to be the ancestral gene, does not group with the *A. thaliana AE7* gene found to participate in the CIA machinery (Figure 6). All angiosperms have at least one *AE7* gene. Genes forming the third clade are found specifically in Brassicaceae suggesting that an additional duplication occurred in the last common ancestor of this plant family. Altogether, these observations raise the question of the involvement of the ancestral gene in Fe-S cluster assembly and of the role of additional genes in organisms having multiple paralogs.

The SUFE family has been expanded in plants and its evolution in the green lineage is particular. The ancestral gene, found in cyanobacteria, encodes a single SufE domain protein and it does correspond to the *SUFE2* gene found in dicots. Gene fusion with a *BOLA* gene (for *SUFE1*) or with a quinolinate synthase *NADA* gene (for *SUFE3*) probably occurred in the ancestor of green algae and has been conserved throughout evolution in accordance with the observation that they are indispensable for plant physiology or development (Xu and Moller, 2006; Ye et al., 2006a; Murthy et al., 2007). Interestingly, a *SUFE2* gene is only found in dicots, except poplar (Figure 6). Due to its specific expression in pollen, this gene is probably associated with Fe-S cluster biogenesis specifically in this organ (Murthy et al., 2007). It may have been replaced by another SUFE protein in monocot flowers and became dispensable in green algae, or non-flowering organisms as *Selaginella* and *Physcomitrella*.

Concerning the NFU family, cyanobacteria have a single protein (NfuA) consisting of a single Nfu domain, whereas eukaryotes have usually two NFU prototypes corresponding to the plastidial NFU1-3 type and to the mitochondrial NFU4-5 type. The NFU1-3 type is typically formed by two Nfu domains, an N-terminal one that conserved the cysteine residues involved in Fe-S cluster binding and a degenerate C-terminal domain without these cysteines. Hence, these genes likely result from a fusion event between two *NFU* genes that took place in the last common ancestor of green algae. The NFU4-5 type is formed by a domain of unknown function of about 90 amino acids at the N-terminus and a single Nfu domain at the C-terminus. Hence, these genes have probably been formed through a different gene reshuffling event compared to *NFU1-3*. Most algae possess two genes of the NFU1-3 type and one gene of the NFU4-5 type, meaning that additional duplication events occurred in some angiosperms, in particular in *A. thaliana* (Figure 6). Very interestingly, one of the two NFU1-3 proteins present in some algae possesses, in addition to the two Nfu domains, an N-terminal extension of about 200 amino acids, with a predicted GIY-YIG nuclease domain. In this fusion protein, the Nfu domain may serve as a scaffold for the coordination of a divalent metal ion required for catalysis of the nuclease domain. This kind of sequence is also found in *P. patens* and in *Picea sitchensis*, while it seems to be absent in *S. moellendorffii* and in all other angiosperms analyzed. In this case, the evolutionary scenario is more uncertain. Even more interesting, this domain displays some similarity to the one found at the N-terminus of GRXS16, a Grx isoform specific to photosynthetic organisms. However, the similarity between the nuclease domains of these two proteins is not that strong, which may be explained by the existence of two different ancestral genes in cyanobacteria. Altogether, although the activity and importance of these N-terminal regions will have to be explored, this suggests a functional relationship between the two domains.

With the same idea, we have also noted that HCF101 proteins possess an N-terminal DUF59 domain that is also found in AE7 proteins, suggesting a conserved function of this domain possibly for protein-protein interactions and maybe for the recognition of proteins containing [Fe_4_S_4_] clusters (Lezhneva et al., 2004; Schwenkert et al., 2010; Luo et al., 2012).

A last intriguing case concerns *IBA57* genes. Most photosynthetic eukaryotes possess one *IBA57.1* gene related to those of α-proteobacteria and one *IBA57.2* gene related to those of cyanobacteria, coding for mitochondrial and plastidial proteins, respectively. Surprisingly, monocots and poplar do not possess the *IBA57.2* gene raising questions about the role and importance of the plastidial isoform for plants. In *A. thaliana*, the mitochondrial isoform is indispensable while no mutant for the *IBA57.2* gene was characterized so far (Waller et al., 2012). As proposed in this study, it is, however, possible that in organisms lacking IBA57.2, IBA57.1 is targeted to both sub-cellular compartments.

## Current open questions

In this part, we focus on a number of questions about key steps in the assembly process which are the subject of intense research or which should receive attention in the future.

### Where do iron atoms come from?

As reported above, the process of sulfur mobilization for Fe-S cluster assembly is well-described and involves cysteine desulfurases conserved throughout kingdoms. In contrast, the origin of iron is still largely unknown. As sulfide and iron are toxic as free entities, the cell has to tightly regulate the intracellular concentrations of both atoms. Moreover, whether there is a sequential or combined delivery of iron and sulfide on scaffold proteins for building an Fe-S cluster is under intense investigation.

The Fe storage proteins, ferritins, which are conserved in most organisms, would have been an obvious iron donor candidate both for heme and Fe-S cluster synthesis, but there is no evidence supporting their involvement (Briat et al., 2010). In *Arabidopsis*, a triple mutant (*atfer1-3-4*) for leaf ferritins has no apparent phenotype (Ravet et al., 2009), whereas mutant plants altered in most biogenesis factors are lethal. Moreover, frataxin-deficient yeast cells expressing the human mitochondrial ferritin still accumulate excess iron in their mitochondria and remained deficient in Fe-S cluster assembly (Sutak et al., 2012).

Much more attention has been paid to frataxin. The *E. coli* CyaY ortholog was shown to interact with the IscU–IscS complex *in vitro* (Adinolfi et al., 2009) and to bind iron with low affinity (Bou-Abdallah et al., 2004; Layer et al., 2006), without involving histidine or cysteine which are classical conserved residues found in iron-binding proteins (Bou-Abdallah et al., 2004; Pastore et al., 2007). It was also reported that the CyaY protein decreased the activity of the IscS cysteine desulfurase, leading to inhibition of the Fe-S cluster assembly (Prischi et al., 2010; Iannuzzi et al., 2011).

The yeast frataxin homologue (Yfh1) interacts physically with both Nfs–Isd11 and Isu1 (Gerber et al., 2003), it binds iron with high affinity (Stemmler et al., 2010; Subramanian et al., 2011) and it is required early in the ISC pathway for Fe-S cluster assembly on Isu1 (Mühlenhoff et al., 2003; Hoff et al., 2003). However, no clear consensus has been reached so far on the mechanistic role of this protein, although it is well-accepted that its primary function in yeast and human mitochondria is in Fe-S protein biogenesis. Frataxin is required for Nfs1–Isd11 desulfurase activity during *in vitro* Fe-S cluster synthesis (Tsai and Barondeau, 2010), but its function is not essential and serves to improve the efficiency of the ISC machinery (Yoon et al., 2012). A direct transfer of frataxin-bound iron to acceptor proteins has been documented (Yoon and Cowan, 2003), but an unambiguous *in vivo* confirmation has not been obtained so far (Mühlenhoff et al., 2002). Very recently, the biochemical and spectroscopic analyses of mouse Nfs1-IscU-Isd11 complexes with or without frataxin indicated that frataxin could control iron entry in the quaternary complex through the activation of Nfs1 cysteine desulfurase activity, at least in mammals (Colin et al., 2013).

Only one frataxin gene coding for an essential mitochondrial protein is found in the *Arabidopsis* genome (Busi et al., 2004). Indeed, contrary to yeast frataxin or *E. coli cyay* mutants which are perfectly viable, a KO mutant is embryo-lethal (Vazzola et al., 2007). A knockdown mutant in *A. thaliana* displayed a strong decrease of the activity of two mitochondrial Fe-S enzymes, namely aconitase and succinate dehydrogenase (Busi et al., 2006). The *atfh-1* plants also present a 1.6 fold elevated total iron content comparatively to wild type plants. It reflects a mitochondrial and possibly plastidial Fe content increase (Martin et al., 2009). Consequently, these plants, which have increased levels of superoxide and other ROS, are also hypersensitive to oxidative stress. Moreover, the analysis of these plants reveals an increase in NO production which helps to maintain low levels of oxidative damage in root cells, concomitant with the induction of the expression of the *FER1* and *FER4* ferritin genes (Martin et al., 2009). The role of plant frataxin in mitochondrial iron homeostasis has recently been reinforced by the demonstration of its involvement in heme synthesis (Maliandi et al., 2011). However, the precise biochemical function of frataxin remains to be determined.

Additional candidates for the iron-delivery function are ATC proteins and Grxs. Several arguments in favor or against the involvement of both proteins can be found in the section (The CIA Machinery in Yeast and Mammals) for Grxs and in the next section (Functional Analysis of NFU and ATC Proteins: How to Differentiate Between Iron Donor, Scaffold or Carrier Functions?) for ATCs.

### Functional analysis of NFU and ATC proteins: how to differentiate between iron donor, scaffold or carrier functions?

In the current literature, there is very often confusion between scaffold and carrier functions. Basically, a scaffold protein is the primary site of *de novo* cluster assembly. It interacts directly with the cysteine desulfurase or the persulfide-carrying partner protein (typically SufE) and most of the time it stimulates cysteine desulfurase activity. In contrast, carrier proteins would rather interact with the scaffold proteins or eventually with the chaperones also required for the cluster trafficking. Carrier proteins can generally receive Fe-S clusters from scaffold proteins but the opposite is not true. While it is generally accepted that IscU and SufBCD are scaffold proteins, the exact role of many other components, especially NFU and ATC proteins is not clearly defined.

Similar to human Nfu1 isoform, bacterial NfuA proteins from *E. coli*, *A. vinelandii* and *Synechococcus* sp. PCC 7002 can form [Fe_4_S_4_] clusters upon reconstitution into dimeric proteins and they are able to transfer *in vitro* their cluster to various acceptor apoproteins, aconitase and PsaC for the cyanobacterial isoform (Tong et al., 2003; Angelini et al., 2008; Bandyopadhyay et al., 2008b; Jin et al., 2008; Py et al., 2012). While Nfu is an essential gene in *Synechococcus* and in human (Balasubramanian et al., 2006; Navarro-Sastre et al., 2011), this is not the case in *A. vinelandii* and *E. coli* since the mutants are viable, although an *A. vinelandii* NfuA mutant becomes lethal under elevated oxygen concentrations (Angelini et al., 2008; Bandyopadhyay et al., 2008b). In *A. thaliana*, inactivating *NFU2* gene resulted in a dwarf phenotype, primarily explained by an impaired PSI accumulation and thus deficient photosynthesis (Touraine et al., 2004; Yabe et al., 2004). A very detailed biochemical and functional analysis was achieved for *E. coli* NfuA, showing that both the degenerate ATC N-terminal domain, which is responsible of protein substrate recognition, and the Nfu domain are important for its *in vivo* function (Py et al., 2012). Considering the definitions detailed above, *E. coli* NfuA is definitely defined as a carrier protein since it has no effect on cysteine desulfurase activities and does not interact with cysteine desulfurases, whereas it can accept an Fe-S cluster from IscU/HscBA or SufBCD scaffold complex proteins (Py et al., 2012).

Concerning ATC proteins, their physiological functions are subject to an intense debate because their *in vitro* biochemical properties (type of bound Fe-S clusters and oligomeric state) do not entirely match *in vivo* analyses. *A. vinelandii* ^Nif^IscA, and *Erwinia chrysanthemi* SufA are homodimeric proteins binding both labile [Fe_2_S_2_] and [Fe_4_S_4_] clusters, whereas *E. coli* IscA and SufA and *A. thaliana* SUFA1 binds [Fe_2_S_2_] clusters, the former being tetrameric and the two latter dimeric (Ollagnier-de Choudens et al., 2003, Ollagnier-de-Choudens et al., 2004; Cupp-Vickery et al., 2004; Abdel-Ghany et al., 2005; Gupta et al., 2009; Mapolelo et al., 2012b). All Fe-S cluster-bound ATCs can generally efficiently transfer their clusters to usual acceptor proteins and some ATC can reversibly convert between [Fe_2_S_2_]^2+^ and [Fe_4_S_4_]^2+^ forms, which is convenient for delivering the correct type of clusters to specific proteins. Besides, several ATCs also bind mononuclear iron with different affinities raising the question of its *in vivo* significance (Cupp-Vickery et al., 2004; Sendra et al., 2007; Lu et al., 2010; Mapolelo et al., 2012a). The demonstration that ATCs do not interact with the cysteine desulfurase systems and that they can accept clusters formed on primary scaffold proteins, but that the opposite in not true, led to define them as carrier proteins. This has been clearly demonstrated for *E. coli* IscU/IscA, *E. coli* SufBCD/SufA or *A. vinelandii* NifU/^Nif^IscA couples (Ollagnier-de-Choudens et al., 2004; Chahal and Outten, 2012; Mapolelo et al., 2012b). Thus, the current view is that ATC proteins are very versatile, binding mononuclear iron and accepting *in vitro* both types of preassembled clusters from primary scaffolds, and transferring also both types to acceptor proteins. However, the study of organisms depleted for *ATC* genes may help to differentiate between both capacities. Depletion of the mitochondrial Isa1/Isa2 in *S. cerevisiae* and ISCA1/ISCA2 in human or of IscA/SufA in *E. coli* indicated that they are not essential genes and that the proteins are involved in the maturation of [Fe_4_S_4_]proteins but not of [Fe_2_S_2_]proteins (Tan et al., 2009; Mühlenhoff et al., 2011; Sheftel et al., 2012). Similarly, deletion of SufA or IscA in prokaryotes is usually neither detrimental nor lethal, unless mutations are combined or under specific conditions, and depletion of the chloroplastic SUFA1 in *A. thaliana* has no effect when plants are grown under control conditions (Balasubramanian et al., 2006; Yabe and Nakai, 2006; Lu et al., 2008). These observations are not in favor of a role as a general Fe donor since the mutation of such genes would be expected to be lethal.

### How is Fe-S cluster biogenesis regulated/coordinated at the cellular and plant scale and by which proteins/molecules?

The role of *A. thaliana* ATM3 and the whole ISC export machinery in the cross-talk between the mitochondrial ISC system and the cytosolic CIA system has already been discussed previously. Similarly, an involvement of NEET proteins for the regulation of inter-organellar Fe or Fe-S distribution has been proposed but it awaits firm confirmation.

#### Transcriptional control in response to environmental factors

Fe-S clusters are sensitive to oxygen and some derived reactive species such as superoxide anion, hydrogen peroxide or nitric oxide and their synthesis should be affected by iron and sulfur limitations. Hence, variations of several environmental factors should undoubtedly modulate the functioning of the biogenesis systems but this has been poorly documented to date.

In bacteria, many reports indicate that, when both systems are present, the ISC system behaves as the house-keeping machinery, whereas the SUF system is rather expressed under stress conditions (Lee et al., 2004; Outten et al., 2004; Yeo et al., 2006). In *E. coli*, the expression of both machineries is controlled by the IscR transcriptional regulator, which exists under two forms. An holo-IscR form containing a [Fe_2_S_2_] cluster has the ability to bind to the promoter of the *isc* operon, repressing its expression (Schwartz et al., 2001). Impairment or loss of its cluster, due to sensitivity to oxygen or iron depletion, converts IscR to an apo-form which is released from the *isc* promoter, leading to the transcriptional activation of the *isc* operon. Incidentally, apo-IscR will activate the *suf* operon through its binding to the *suf* promoter region (Giel et al., 2006; Yeo et al., 2006). Therefore, under conditions of iron limitation and/or oxidative stress, the expression of both the ISC and SUF systems is induced. However, the SufS-SufE couple is less susceptible to H_2_O_2_-mediated oxidation than the IscS-IscU couple (Dai and Outten, 2012) and the SUF system may rely on an iron-independent flavin reductase system for electron donation instead of an iron-dependent system (FdR-Fdx) for the ISC system. Therefore, the SUF system is likely more adapted to oxidative conditions. It is notable that the *suf* and *isc* operons are also controlled by the OxyR, IHF and Fur transcription factors, but the interplay between all these regulatory mechanisms remains to be precisely investigated. In addition to this transcriptional control, the ISC system is also regulated by a small non-coding RNA, RyhB (Desnoyers et al., 2009). In response to Fe deficiency, RyhB is expressed and it binds to *iscS*, the second cistron of the polycistronic *iscRSUA* mRNA, leading to the cleavage of the downstream *iscSUA* transcript. A model of the genetic regulation of Fe-S cluster assembly systems in *E. coli* can be found in (Roche et al., 2013).

There is growing evidence that some assembly factors, especially carrier proteins, as ErpA and NfuA, are linked to both machineries in *E. coli* and that there is a certain level of redundancy (Py et al., 2011). However, the current view is that there may be different trafficking pathways for a single protein depending on the conditions. Typically the genetic analysis of null mutants for some genes indicated that lethal phenotypes are only observed under specific conditions. For instance, a very nice example concerns the maturation of the IspG and IspH proteins in *E. coli*, two enzymes required for the synthesis of isopentenyl diphosphate. It would necessitate the SufBCD–SufA system under stress conditions, but would use IscU, IscA and then ErpA in this order under aerobic conditions, and IscU and either ErpA or IscA in anaerobic conditions (Vinella et al., 2009).

In eukaryotic cells, and in yeast in particular, the regulation of expression of the genes encoding the various Fe-S cluster biogenesis machineries has not been studied into detail. It is so far unknown if the coordination of their action is mediated through transcriptional control, as in prokaryotes. As an example, it was shown that human *ISCU1/2* are repressed during hypoxia by the microRNA-210, as a way to control mitochondrial metabolism under this condition (Chan et al., 2009).

In plants, although public databases for gene expression can be questioned, almost no report has been published concerning the regulation of the expression of the ISC, SUF or CIA components. None of the above reported transcriptional regulators are encoded in plant or algal genomes. Some studies on isolated genes have been performed showing for example that in *A. thaliana*, *SUFB* gene expression is repressed upon iron starvation but the regulatory mechanisms are not known (Xu et al., 2005). More detailed studies would be important, in particular in the case of the SUF system which is located in the chloroplast, and therefore exposed to important changes in O_2_ concentration between day and night, and to oxidative stress under high light intensity conditions. The regulation of expression of the genes encoding the SUF system has been investigated in *Synechocystis* sp. PCC 6803 (Wang et al., 2004), a cyanobacterium with an evolutionary relationship to chloroplasts. A SufR gene regulator possesses a DNA-binding domain and exhibits four highly conserved cysteine residues near its C-terminus enabling the coordination of two [Fe_4_S_4_] cluster (Shen et al., 2007). Cells grown under oxidative or iron stress conditions have elevated levels of expression of the Suf operon, which are even higher in a null *sufR* mutant. SufR acts therefore as a transcriptional repressor whose activity depends on the presence or absence of an Fe-S cluster. Other information came from work done with the cyanobacterium *Synechococcus* PCC7002. In this organism, it has been proposed that, instead of having direct roles for Fe-S cluster assembly, SufA and IscA could play regulatory roles in iron homeostasis and in the sensing of redox stress (Balasubramanian et al., 2006). Clearly, there is a pressing need to understand whether and how assembly factors are controlled at the transcriptional level. Important conditions to assess would be those leading to iron and sulfur starvation, iron excess, and several other environmental constraints known to generate reactive oxygen or nitrogen species. Besides, the regulation of these genes in plants grown under various regimes of temperature and photoperiod or in response to a light/dark cycle would also bring valuable information to better understand the mechanisms of *de novo* synthesis or repair of Fe-S clusters.

#### Post-translational control: does the Grx/bola interaction also constitute a regulatory link between Fe-S cluster biogenesis and cellular iron regulation in plants?

BolA has been initially identified in a screen for *E. coli* mutants with altered cell morphology, which in the case of *bolA* mutation results in a round cell shape morphology (Aldea et al., 1988). In addition, BolA seems to be involved in cell protection from stress, cell proliferation or cell-cycle regulation (Kim et al., 2002). More recently, it was demonstrated that human BolA3 is required for the maturation of lipoate-containing 2-oxoacid dehydrogenases and for the assembly of the respiratory chain complexes (Cameron et al., 2011).

In *S. cerevisiae*, genetic and biochemical studies have revealed that Grx3 and Grx4 form a complex with two other proteins named Fra1 and Fra2 (Fe repressor of activation-1 and 2) corresponding to an aminopeptidase P-like protein and a BolA protein, respectively (Kumanovics et al., 2008). By interacting with the transcription factor Aft1p, which controls the expression of iron uptake and storage genes in *S. cerevisiae*, this complex senses the status of the mitochondrial Fe-S cluster biogenesis and regulates in turn Aft1 nuclear localization (Ojeda et al., 2006; Pujol-Carrion et al., 2006; Kumanovics et al., 2008).

However, Aft1 proteins are only present in a specific group of yeast species. For instance, in *Schizosaccharomyces pombe*, iron homeostasis is controlled by other transcription factors as Fep1 and Php4 and their activity is also regulated through a direct interaction with cytosolic monothiol Grxs but a requirement of BolA has not been explored (Mercier and Labbe, 2009; Jbel et al., 2011; Kim et al., 2011). As exemplified for the interaction with Aft1, it has been proposed that the C-terminal region of cytosolic monothiol Grxs constitutes the binding site for most iron responsive transcriptional factors, at least in fungi (Hoffmann et al., 2011).

Based on these observations, it appears crucial to explore whether the Grx-BolA proteins also participate to an iron sensing mechanism in other organisms. First, bioinformatic analyses indicated that both genes are frequently adjacent in prokaryotic genomes that some natural fusion proteins exist in a few microbes and that there is a very strong gene co-occurrence of these genes (Couturier et al., 2009). Moreover, high-throughput screen for interaction partners using yeast two hybrid studies showed that *Drosophila*, yeast and plant Grx and BolA proteins interact (Ho et al., 2002; Giot et al., 2003; Braun et al., 2011) and several biochemical studies have demonstrated that Grx and BolA from *S. cerevisiae*, *E. coli* and human can form [Fe_2_S_2_]-bridged heterodimers (Li et al., 2009, 2011, 2012; Yeung et al., 2011). In addition, recent work on the human mitochondrial BolA1 isoform revealed that it can interact with the mitochondrial Grx5 (Willems et al., 2013).

In plants, genomic analysis revealed the existence of four genes coding for proteins containing a BolA domain. Interestingly, whereas three of these genes code for proteins with a single domain and whose function is unknown, plants possess the chloroplastic SUFE1 protein which is a fusion protein constituted by an N-terminal SufE domain and a C-terminal BolA domain. As the activity of the cysteine desulfurase NFS2 is increased by SUFE1 (Xu and Moller, 2006; Ye et al., 2006a), it is tempting to speculate that plastidial monothiol Grxs could regulate the functioning of the SUF machinery by controlling the SUFE1-dependent NFS2 activity through an interaction with the BolA domain of SUFE1. Another possible regulatory mechanisms would consist of redox-dependent post-translational control of the sulfurtransferase activity as both *E. coli* SufE and CsdE, which do not have a BolA domain, have been shown to interact with a monothiol Grx through an intermolecular disulfide bond (Bolstad et al., 2010; Bolstad and Wood, 2010). Finally, using *in vitro* Fe-S cluster transfer experiments, it was shown that A-type proteins were able to transfer an [Fe_2_S_2_] cluster to a Grx-BolA heterodimer, whereas, in the absence of BolA, the only cluster transfer observed was in the opposite direction, i.e., from cluster-bound holodimeric forms of glutaredoxins to A-type carriers (Mapolelo et al., 2013). This led to the conclusion that BolA proteins could convert monothiol Grxs from cluster donors to net cluster acceptors. All these data point to the possible involvement of Grx-BolA complexes either in the regulation of Fe-S cluster biogenesis or in the sensing of Fe-S cluster status in organelles where both proteins are simultaneously present.

#### Are there Fe-S cluster repair mechanisms?

Fe-S clusters in most proteins/enzymes can readily and directly react with oxygen and its derived oxidant molecules as peroxynitrite, superoxide ions, hydrogen peroxide, leading both to enzyme inactivation and to the release of Fe^2+^ (Keyer and Imlay, 1997; Djaman et al., 2004; Imlay, 2006; Jang and Imlay, 2007). This loss can fuel Fenton chemistry, producing even higher levels of toxic reactive oxygen species, which are deleterious to most macromolecules. This underlines quite simply the futile cycle that could occur upon metal release during oxidative stress conditions. This aspect is particularly important to consider in the context of the chloroplast, a sub-cellular compartment producing important levels of reactive oxygen or nitrogen species especially under environmental constraints. Thus, how cells maintain the activity of enzymes with sensitive clusters is a crucial question, because of the essential nature of many Fe-S enzymes for the cellular functioning. The simplest view is that cells, and plastids in particular, need more robust biogenesis pathways, and/or Fe-S reservoirs and/or repair systems. A few *in vivo* experiments have highlighted the existence of repair systems for damaged Fe-S proteins. This was illustrated for dehydratases whose [Fe_4_S_4_] clusters are rapidly degraded into [Fe_3_S_4_] forms in contact with univalent oxidants as hydrogen peroxide, superoxide or peroxynitrite (Keyer and Imlay, 1997; Djaman et al., 2004). By assessing in whole cells the disappearance of [Fe_3_S_4_] EPR signal upon scavenging of hydrogen peroxide, it was concluded that *in vivo* repair systems exist. In bacteria, several genetic or biochemical evidence showed that the bacterial di-iron proteins YtfE belonging to the RIC family, and some ferritins as FtnA or FtnB or the YaaA protein could transiently store released iron (Velayudhan et al., 2007; Bitoun et al., 2008; Overton et al., 2008; Liu et al., 2011). This mechanism could attenuate the Fenton reaction that would occur in the presence of higher intracellular iron levels and could facilitate the re-assembly of the disrupted iron-sulfur clusters. In line with these results, it was found that the iron stored in FtnA can be retrieved by IscA for the re-assembly of the iron-sulfur cluster in the IscU scaffold protein (Bitoun et al., 2008).

As mentioned previously, additional observations open new perspectives in the understanding of cluster disassembly/reassembly process in particular for O_2_-sensitive [Fe_4_S_4_] clusters and *in vivo* repair mechanisms. Indeed, a cysteine persulfide-coordinated [Fe_2_S_2_] cluster has been observed in several enzymes as FNR, and most likely in aconitase or biotin synthase upon O_2_-mediated disassembly of [Fe_4_S_4_] (Kennedy and Beinert, 1988; Zhang et al., 2012). For FNR, this intermediate can be used for the re-assembly of an active [Fe_4_S_4_] cluster pending the presence of iron and a reducing agent. These results have been strengthened by the recently described 3D structure of an [Fe_2_S_2_] cluster bound by two cysteine persulfides in the hydrogenase maturase HydE from *Thermotoga maritima* (Nicolet et al., 2013).

## Conclusions and perspectives

The assembly of cellular Fe-S clusters in plants is governed by three biogenesis systems, the SUF, ISC and CIA machineries. Contrary to the plastidial SUF machinery which seems to function independently, the maturation of cytosolic and nuclear Fe-S proteins *via* the CIA machinery requires the function of the mitochondrial ISC machinery through an export system likely providing the sulfur source. Whereas the source of sulfur and of electrons required for these machineries has been *a priori* elucidated, how iron is mobilized and delivered is very poorly understood. Moreover, the current knowledge on the precise roles of each identified component is still limited and there is a pressing need to understand the functions of the remaining participants. In particular, whether GRX, ATC and NFU proteins act as scaffold or carrier proteins or eventually as iron donors has not been firmly elucidated so far. It is worth noting that many studies in plants are complicated by the fact that several genes are essential for embryo development. Hence, improving our knowledge on these mechanisms will require the study of knock down or conditional mutants as successfully achieved for AtNFS2 (Van Hoewyk et al., 2007). Another under-explored area of research concerns the regulation of the assembly processes by environmental factors and constraints and the repair mechanisms of damaged Fe-S clusters. A recent study highlighted that a circadian-controlled retrograde pathway from plastid-to-nucleus and involving phytochromes participates in Fe sensing (Salome et al., 2013). This opens interesting perspectives to decipher the signaling pathways and regulatory mechanisms allowing plant cells to adapt to changing conditions.

### Conflict of interest statement

The authors declare that the research was conducted in the absence of any commercial or financial relationships that could be construed as a potential conflict of interest.
